# Comparison of self-report versus accelerometer – measured physical activity and sedentary behaviors and their association with body composition in Latin American countries

**DOI:** 10.1371/journal.pone.0232420

**Published:** 2020-04-28

**Authors:** Gerson Luis de Moraes Ferrari, Irina Kovalskys, Mauro Fisberg, Georgina Gómez, Attilio Rigotti, Lilia Yadira Cortés Sanabria, Martha Cecilia Yépez García, Rossina Gabriella Pareja Torres, Marianella Herrera-Cuenca, Ioná Zalcman Zimberg, Viviana Guajardo, Michael Pratt, Carlos André Miranda Pires, Rachel C. Colley, Dirceu Solé, on behalf of the ELANS Study Group

**Affiliations:** 1 Centro de Investigación en Fisiologia del Ejercicio - CIFE, Universidad Mayor, Santiago, Chile; 2 Departamento de Pediatria da Universidade Federal de São Paulo, São Paulo, Brazil; 3 Commitee of Nutrition and Wellbeing, International Life Science Institute (ILSI-Argentina), Buenos Aires, Argentina; 4 Instituto Pensi, Fundação José Luiz Egydio Setubal, Hospital Infantil Sabará, São Paulo, Brazil; 5 Departamento de Bioquímica, Escuela de Medicina, Universidad de Costa Rica, San José, Costa Rica; 6 Centro de Nutrición Molecular y Enfermedades Crónicas, Departamento de Nutrición, Diabetes y Metabolismo, Escuela de Medicina, Pontificia Universidad Católica, Santiago, Chile; 7 Departamento de Nutrición y Bioquímica, Pontificia Universidad Javeriana, Bogotá, Colombia; 8 Colegio de Ciencias de la Salud, Universidad San Francisco de Quito, Quito, Ecuador; 9 Instituto de Investigación Nutricional, La Molina, Lima, Peru; 10 Centro de Estudios del Desarrollo, Universidad Central de Venezuela (CENDES-UCV)/Fundación Bengoa, Caracas, Venezuela; 11 Departamento de Psicobiologia, Universidade Federal de São Paulo, São Paulo, Brazil; 12 Institute for Public Health, University of California San Diego, La Jolla, CA, United States of America; 13 Center for Research in Neuropsychology and Cognitive and Behavioral Intervention (CINEICC), Faculty of Psychology and Educational Sciences, University of Coimbra, Portugal; 14 Health Analysis Division, Statistics Canada, Ottawa, Ontario, Canada; Kennesaw State University, UNITED STATES

## Abstract

**Background:**

Most population-based studies from Latin America have used questionnaires to measure physical activity (PA) and sedentary behaviors (SB). Low reliability and validity of the questionnaires has limited the capacity to examine associations between PA and health. The purpose of this study was to compare self-reported and accelerometer–measured PA and SB and their associations with body composition in Latin American countries.

**Methods:**

Data were obtained from the Latin American Study of Nutrition and Health (aged 15–65 years), collected from September 2014 to February 2015. PA and SB were assessed using the International Physical Activity Questionnaire (long version) and the Actigraph GT3X+ accelerometer. Outcomes of interest included: body mass index (BMI), waist (WC) and neck circumference (NC). We used the Pearson and intraclass correlation coefficient, Bland-Altman plots, and multilevel linear regression models.

**Results:**

Mean moderate-to-vigorous physical activity (MVPA) by accelerometer and IPAQ were 34.4 min/day (95% CI: 33.4 to 35.4) and 45.6 min/day (95% CI: 43.2 to 48.1), respectively. For SB (accelerometer and IPAQ) the means were 573.1 (95% CI: 568.2 to 577.9) and 231.9 min/day (95% CI: 225.5 to 238.3). MVPA, measured by the accelerometer was negatively associated with BMI (β = -1.95; 95% CI: -2.83 to -1.08), WC (β = -5.04; 95% CI: -7.18 to -2.89) and NC (β = -1.21; 95% CI: -1.79 to -0.63). The MVPA estimated through IPAQ was not significantly associated with any of the three outcome variables. SB, measured by the accelerometer, was positively associated with BMI (β = 0.26; 95% CI: 0.08 to 0.44) and WC (β = 0.48; 95% CI: 0.13 to 0.91). SB estimated through IPAQ was positively associated with NC only.

**Conclusions:**

Low correlation coefficients were observed for accelerometer-derived and IPAQ-reported estimates of PA and SB. Caution is advised when making comparisons between accelerometer-measured and self-reported PA and SB. Further, studies examining associations between movement and health should discuss the impact of PA and SB measurement methodology on the results obtained.

## Introduction

There are clear signs from population-based surveys that levels of physical activity (PA) are negatively associated with cognitive diseases, cancer, chronic disease, and all-cause mortality [1, 2]. Consistent evidence also shows positive associations between sedentary behaviour (SB) and cardiometabolic health markers, including higher triglycerides, higher body composition indices such as body mass index and waist circumference and poor health outcomes, including cardiovascular disease and mortality [3–5].

Both in high-income and in low- and middle-income countries, such as most Latin American countries, the majority of published research on the relationship between PA and SB and health has used self-reported measures of PA and SB [6, 7]. Indirect measurement (questionnaires) are practical, easy to implement in population surveillance studies, and provide important contextual information about PA and SB [8, 9]. Questionnaires are limited by social desirability bias and recall difficulties [8,9]. The International Physical Activity Questionnaire (IPAQ) is a validated questionnaire and was originally developed to allow for cross-national comparisons [10]. Accelerometers are reliable and valid objective instruments for measuring PA, but more expensive and time-consuming than self-report methods [11, 12]. IPAQ performs at least as well as other self-report questionnaires and has been shown to have weak to moderately (r = 0.17–0.55) significant associations with accelerometer-measured PA among adolescents and adults [10, 13, 14].

The high financial burden and technical expertise required to implement accelerometry in epidemiological survey studies has challenged investigators in low- and middle-income countries [15]. As a result, Latin American professionals have relied on questionnaires to measure PA behaviors [16, 17]. Determining the magnitude and cause and effect relationship between PA or SB and risks of adverse health conditions in people from different countries in Latin America depends on an accurate assessment of these behaviors. Inadequate or inconsistent methods can increase the proportion of errors and mask or alter the actual associations between behaviors and outcomes [18].

PA and SB self-report questionnaires are widely applied, increasing the possibilities for comparisons between studies; however, the imprecise values from self-reported results limit their utility in comparison with objective measures such as accelerometry [19] [13]. This is especially true for understanding associations between PA and SB and health outcomes. Therefore, comparison between accelerometry and self-report and validation of self-report is essential for better understanding the strength of the relationship between self-reported PA and SB and objectively assessed with body composition variables in Latin American countries [13]. The Latin American Study of Nutrition and Health (*Estudio Latinoamericano de Nutrición y Salud* - ELANS) has both self-reported and accelerometer-measured PA and SB, providing an opportunity to compare how these two measures are associated with PA and SB in eight Latin America countries. This is especially relevant in Latin America since the region has very high levels of self-reported physical inactivity (> 39.1%) [6]. One of the aims of ELANS was to compare PA and SB as assessed by self-report versus accelerometer and their associations with body composition in Latin America countries.

## Material and methods

### Study design and participants

ELANS was conducted in eight Latin American countries: Argentina, Brazil, Chile, Colombia, Costa Rica, Ecuador, Peru, and Venezuela. ELANS is a household-based, multi-national, cross-sectional study of urban populations [20]. A standard harmonized protocol was used to collect data across eight countries, and all study personnel underwent comprehensive training to ensure quality and consistency in data collection. Data were collected between 2014 and 2015. Study design, methods, sampling strategy and exclusion criteria have been published in detail [20, 21].

**Fig 1:**
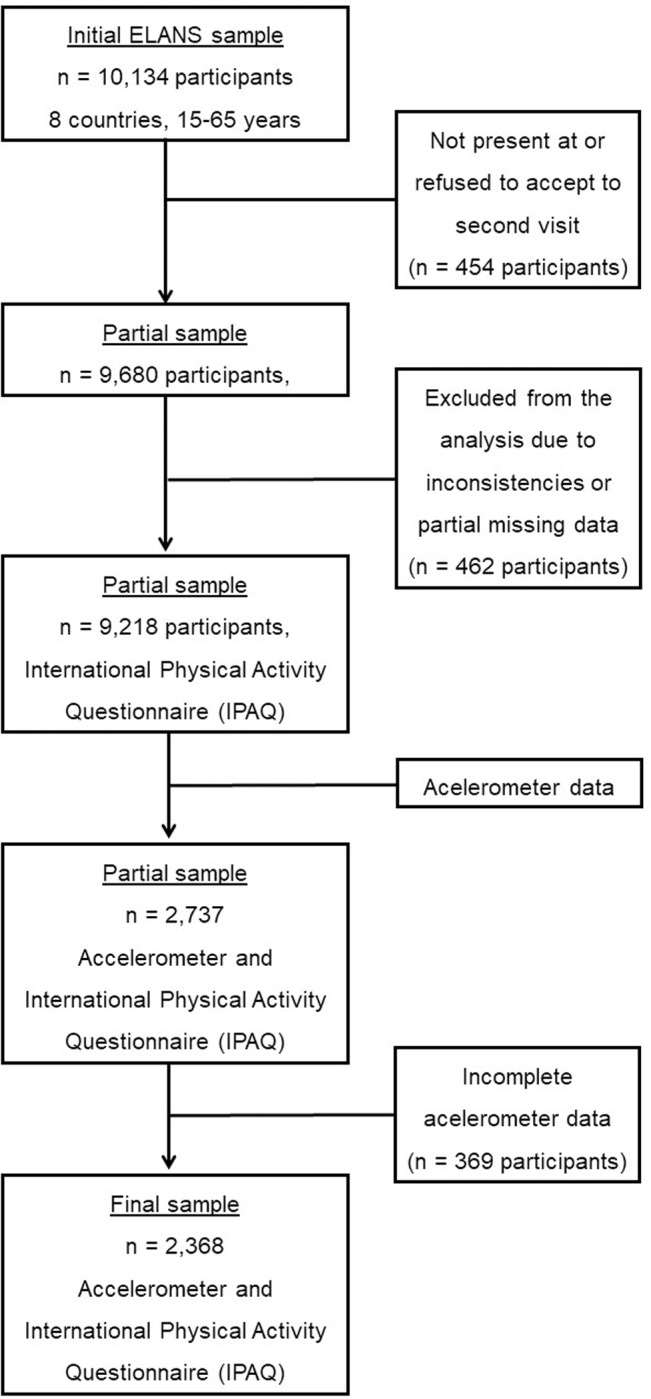
Flow-chart of processes to obtain the final sample.

A total sample of 9,218 (52.2% women) participants (aged 15.0–65.0 years) was included in the ELANS study. The current paper is based on a sample of 2,368 participants aged 15–65 years with valid accelerometer and IPAQ data, representing 29.6% of the total ELANS cohort. Accelerometer assessed PA and SB data were collected in a subsample representative of the total study population by sex, age and socioeconomic level [21]. Overall, the response rate for IPAQ was 99.4% and the response rate for the accelerometer measures was 86.5% of the total accelerometer sample (n = 2,737) (Fig 1).

### Anthropometric data

Body weight (to the nearest 0.1kg) was measured using a portable scale Seca® (Hamburg, Germany) up to 200 kg, after all heavy clothing, pocket items, shoes, and socks were removed [22]. Height was measured with a Seca 213® portable stadiometer (Hamburg, Germany) at the end of a deep inhalation with the participant’s head in the Frankfort Plane whose measuring range was from 0–205 cm [22]. Body mass index in kg/m^2^ was calculated [23].

Neck circumference (in centimeters) was measured at the point just below the larynx (thyroid cartilage), perpendicular to the long axis of the neck (with the tape line in the front of the neck at the same height as the tape line at the back of the neck), using an non-elastic tape measure [24]. Waist circumference was measured (in centimeters) midway between the lowest rib and the iliac crest after normal breath according to World Health Organization (WHO) guidelines, and was conducted on the skin using an non-elastic tape [23, 25]. Two measurements of the anthropometric variables were performed, and the average was used for analysis.

### Assessment of physical activity and sedentary behavior

#### Accelerometer

Mean min/day of moderate-to-vigorous physical activity (MVPA) and SB were measured objectively using the model GT3X+ ActiGraph (Pensacola, FL, USA) accelerometer on an elasticized belt at the right mid-axillary line. The Actigraph GT3X provides reliable and valid estimates of PA [26, 27]. Participants were asked to wear the device while awake and to remove it only when sleeping and during water activities (e.g. showering or swimming). To further ensure protocol compliance, participants filled in an accelerometer log sheet that noted the start- and end-time of use per day. The minimal amount of accelerometer day time that was considered acceptable for inclusion in the sample was 5 days (including at least 1 weekend day) with at least 10 hours/day of wear time following the removal of sleep time [28, 29]. After exclusion of the nocturnal sleep period, waking non-wear time was defined as any sequence of ≥60 consecutive minutes of 0 activity counts [30].

On the eighth day of data collection, the research team went to each participant’s home to retrieve the accelerometers. The team downloaded the data using the latest version of Actilife software (version 6.0; ActiGraph, Pensacola, FL) available. Data were collected at a sampling rate of 30 Hz in 60 second epochs [31]. SB was defined as all activity at ≤100 activity counts/min, ≥1952–5724 activity counts/min for moderate PA, ≥5725 activity counts/min for vigorous PA, and ≥1952 activity counts/min for MVPA [32].

#### International physical activity questionnaire

PA and SB were assessed using the IPAQ, long version for the last seven days, a validated self-report measurement tool for PA and SB that has been widely used in Latin America [10, 33, 34]. PA was assessed using six IPAQ items on the frequency and duration of MVPA as well as walking in leisure time and the amount of cycling undertaken for transport.

IPAQ PA data are reported as min/day of walking, moderate and vigorous PA. Total time (min/day) and time spent in total PA (i.e., transport and leisure-time) were estimated and used as analysis variables. We analyzed transport PA (walking + bicycle) and leisure PA (walking + moderate + vigorous) together.

Data were analyzed in agreement with the IPAQ scoring protocol (https://sites.google.com/site/theipaq/scoring-protocol). Although walking is a moderate intensity activity by MET value, the IPAQ questionnaire includes walking as a separate activity domain from moderate activity, explicitly excluding walking in the moderate activity questions. Thus, to provide a comparable index to the accelerometer- derived moderate activity measure for analysis, the walking and moderate activity domains from the IPAQ were combined into a single ‘moderate’ activity domain.

We considered sitting time as an indicator of SB. Sitting time was assessed from the question in the long-form IPAQ [10, 35]. Participants were asked to report time spent sitting over the past 7 days, separately for weekdays and weekends. We calculated average sitting time per day (min/day) as follows: (weekday time*5 + weekend time*2)/7 [36].

### Demographic variables

Demographic characteristics including sex, age, socioeconomic level and race/ethnicity were assessed using standard questionnaires during in-person interviews. Age was categorized into the following groups: 15–19, 20–34, 35–49 and 50–65 years. While socioeconomic categorization varied across countries, socioeconomic level was grouped into three broad levels of classification (low, medium, high) for all countries. Race/ethnicity was classified as white, mixed (born of father and mother of different race/ethnicities) and other (black, asian, indigenous, mulatto, and gypsy). Complete data can be found in a previous study [20].

### Ethics statement

The overarching ELANS protocol was approved by the Western Institutional Review Board (#20140605) and is registered at Clinical Trials (#NCT02226627). In addition, each country also obtained approval from their local ethical review boards of participating institutions and all participants provided written informed consent.

### Statistical analysis

Descriptive statistics included means, 95% confidence intervals (95% CI) or frequencies. The Kolmogorov-Smirnov test and histograms were used to check data distribution. Accelerometer and IPAQ values were compared using paired student t-tests. The correlation and agreement between estimates obtained from the two methods were evaluated with Pearson correlation coefficient (r), intraclass correlation coefficient (ICC) and Bland-Altman plots.

Multilevel linear regression models, with country as the 2nd level, were used to study the effect of PA on BMI, on neck circumference, and on waist circumference. The models were adjusted for sex, age, socioeconomic level, and race/ethnicity. Statistical analyses were carried out with SPSS (version 24.0). Bland-Altman plots were built with the packages *Bland Altman Leh* [37] and *ggplot2* [38] available in the software R [39]. A significance level of 5% was considered (p < 0.05).

## Results

The sample included 2,368 participants (women: 51.9%; 95% CI: 50.0 to 53.9; n = 1230) aged from 15–65 years (mean: 36.5; 95% CI: 35.9 to 37.1). About half were classified as having a low socioeconomic level (51.0%; 95% CI: 48.9 to 53.0) and being of mixed race/ethnicity (51.7%: 95% CI: 49.8 to 53.5). The means of body mass index, neck and waist circumference were 26.9 kg/m^2^ (95% CI: 26.7 to 27.1), 35.6 cm (95% CI: 35.5 to 35.8) and 88.4 cm (95% CI: 87.8 to 88.9), respectively. Mean MVPA by accelerometer and IPAQ were 34.4 min/day (95% CI: 33.4 to 35.4) and 45.6 min/day (95% CI: 43.2 to 48.1), respectively. For SB (accelerometer and IPAQ) means were 573.1 (95% CI: 568.2 to 577.9) and 231.9 min/day (95% CI: 225.5 to 238.3), respectively (Table 1). The description of total sample profile by country is presented in S1 Table.

**Table 1:** Descriptive analysis (% or mean, 95% CI) of the sample demographics, body composition and physical activity.

Variables	Sample (% or mean, 95% CI)
N	2,368
Sex (% [95% CI])	
Men	48.1 (46.1 to 50.0)
Women	51.9 (50.0 to 53.9)
Age group (% [95% CI])	
15–19	12.4 (11.1 to 13.7)
20–34	37.2 (35.3 to 39.2)
35–49	28.5 (26.7 to 30.3)
50–65	22.0 (20.2 to 23.7)
Socioeconomic level (% [95% CI])	
Low	51.0 (48.9 to 53.0)
Medium	39.4 (37.4 to 41.4)
High	9.7 (8.5 to 10.8)
Ethnicity (% [95% CI])	
White	34.9 (33.0 to 36.7)
Mixed	51.7 (49.8 to 53.5)
Other	13.4 (12.0 to 14.8)
Body mass index (kg/m^2^)	26.9 (26.7 to 27.1)
Neck circumference (cm)	35.6 (35.5 to 35.8)
Waist circumference (cm)	88.4 (87.8 to 88.9)
Accelerometer (min/day)	
Moderate physical activity	33.7 (32.8 to 34.7)
Vigorous physical activity	0.62 (0.54 to 0.70)
Moderate-to-vigorous physical activity	34.4 (33.4 to 35.4)
Sedentary behaviors	573.1 (568.2 to 577.9)
IPAQ (min/day)	
Moderate physical activity ^(1)^	38.8 (36.6 to 41.0)
Vigorous physical activity	6.9 (6.1 to 7.6)
Moderate-to-vigorous physical activity	45.6 (43.2 to 48.1)
Sedentary behaviors	231.9 (225.5 to 238.3)

M: mean; 95% CI: 95% confidence interval; SD: standard deviation; IPAQ: International physical activity questionnaire.

^**(1)**^ moderate and moderate-to-vigorous physical activity for International Physical Activity Questionnaire include walking and cycling.

**Fig 2:**
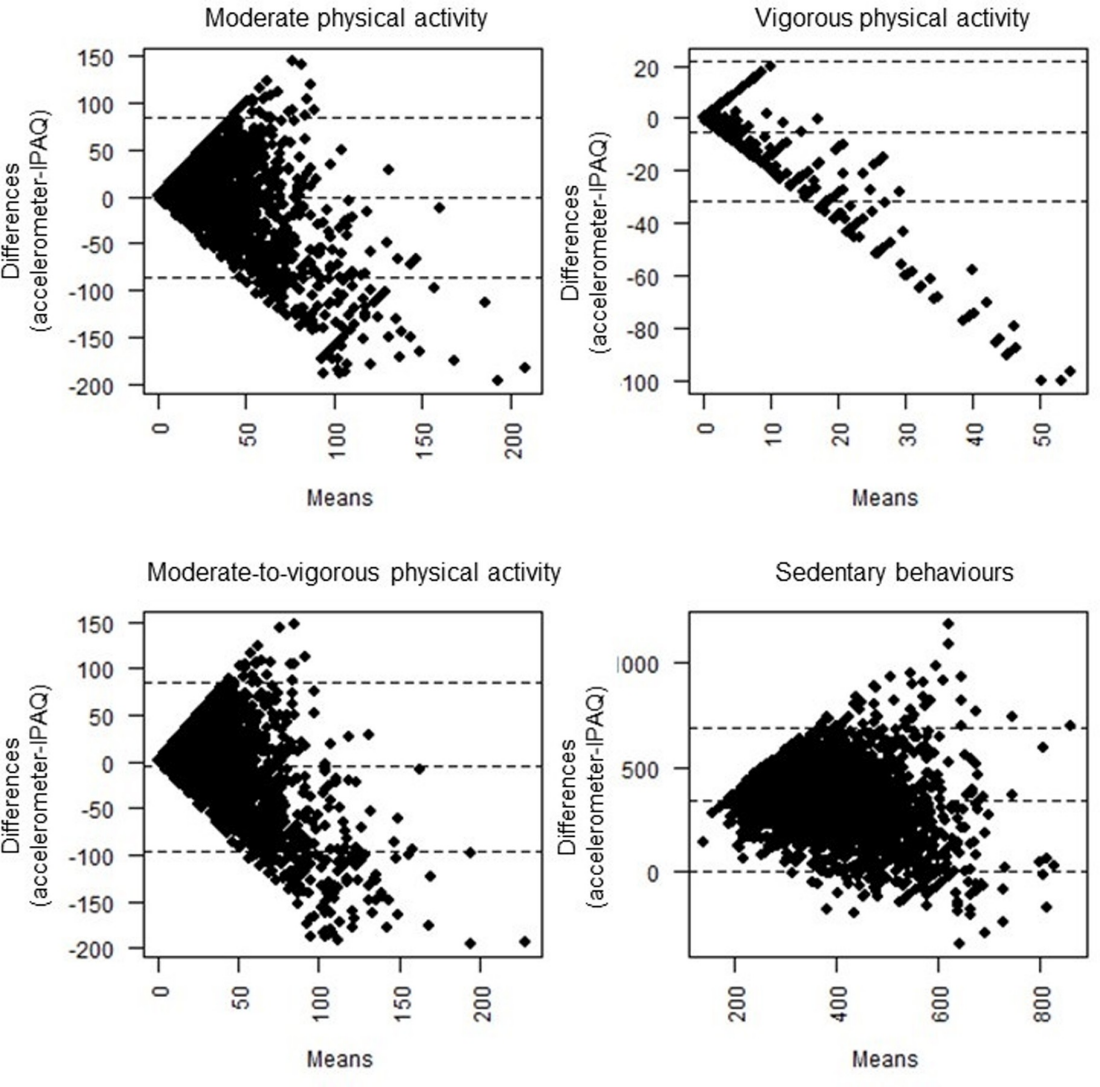
Bland-Altman plots for moderate, vigorous, moderate-to-vigorous physical activity and sedentary behaviors (min/day).

Mean min/day of PA (moderate, vigorous, and MVPA) estimated from IPAQ were significantly higher than those obtained by accelerometry (p<0.001). Correlation coefficients (r = 0.192 for moderate PA, r = 0.180 for vigorous PA, and r = 0.224 for MVPA), the ICC (ICC = 0.247 for moderate PA, ICC = 0.073 for vigorous PA, and ICC = 0.269 for MVPA), and the Bland-Altman plots (Fig 2) show low correlations and low agreement between the minutes of PA obtained by accelerometry and IPAQ. Similar results were found for SB (p<0.001, r = 0.184, and ICC 0.304) (Table 2). Fig 2 shows that differences are similarly distributed for moderate and vigorous PA (mainly negative values), while they were more unilateral for SB (mainly positive values).

**Table 2:** Comparison between accelerometer and IPAQ indices of physical activity and sedentary behaviors.

Accelerometer and IPAQ activity measure	Accelerometer	IPAQ	Difference (IPAQ–accelerometer)	p-value ^(2)^	r	ICC (95% CI)
	Mean (95% CI)	Mean (95% CI)	Mean (95% CI)			
Moderate physical activity ^(1)^	33.7 (32.8 to 34.7)	38.8 (36.6 to 41.0)	5.1 (2.8 to 7.2)	<0.001	0.192	0.247 (0.184 to 0.305)
Vigorous physical activity	0.62 (0.54 to 0.70)	6.86 (6.13 to 7.59)	6.24 (5.52 to 6.95)	<0.001	0.180	0.073 (-0.005 to -0.145)
Moderate-to-vigorous physical activity	34.4 (33.4 to 35.4)	45.6 (43.2 to 48.1)	11.2 (8.7 to 13.6)	<0.001	0.224	0.269 (0.208 to 0.326)
Sedentary behavior	573.1 (568.2 to 577.9)	231.9 (222.5 to 238.3)	-341.2 (-348.2 to -334.0)	<0.001	0.184	0.304 (0.246 to 0.358)

IPAQ: International physical activity questionnaire; ICC: intraclass correlation coefficient; 95% CI: 95% confidence interval; MVPA: moderate-to-vigorous physical activity.

^**(1)**^ Moderate and moderate-to-vigorous physical activity for international physical activity questionnaire include walking and cycling.

^**(2)**^ p-value for comparison between accelerometer and IPAQ mean values (paired Student t-test).

Table 3 shows results of the multilevel regression models for the effects of MVPA (accelerometer and IPAQ) on body mass index, neck circumference and waist circumference. The coefficient (β) represents the change in the dependent variables per 100-minute change in moderate-to-vigorous PA. Results show that MVPA, measured by accelerometer, has a negative effect on the three variables (p<0.001)–an increase of 100 minutes per day of MVPA decreases the body mass index by 1.9 kg/m^2^ (95% CI: -2.8 to -1.1), neck circumference by 1.2 cm (95% CI: -1.8 to -0.6) and waist circumference by 5.0 cm (95% CI: -7.2 to -2.9), independent of sex, age, socioeconomic level, and ethnicity. MVPA estimated from IPAQ was not significantly associated with any of the three variables (p>0.05).

**Table 3:** Multilevel linear regression models for the effect of moderate-to-vigorous physical activity on body mass index, on neck circumference, and on waist circumference.

Dependent variables	Moderate-to-vigorous physical activity (accelerometer)		Moderate-to-vigorous physical activity (IPAQ) ^(1)^	
	β (95% CI)	p-value	β (95% CI)	p-value
Body mass index (kg/m2)	-1.95 (-2.83 to -1.08)	< 0.001	-0.29 (-0.63 to 0.06)	0.102
Neck circumference (cm)	-1.21 (-1.79 to -0.63)	< 0.001	-0.11 (-0.34 to 0.12)	0.337
Waist circumference (cm)	-5.04 (-7.18 to -2.89)	< 0.001	-0.83 (-1.67 to 0.01)	0.052

Multilevel linear regression models, with country as 2nd level, adjusted for sex, age, socioeconomic level, and ethnicity.

β: represents the change in risk factor (body mass index, neck circumference and waist circumference) per 100-minute change in moderate-to-vigorous physical activity.

^(1)^ moderate and moderate-to-vigorous physical activity for international physical activity questionnaire include walking and cycling.

SB, measured by accelerometer was significantly associated with body mass index and waist circumference (p<0.005) - an increase of 100 minutes per day of SB increases the body mass index by 0.3 kg/m^2^ (95% CI: 0.1 to 0.4) and the waist circumference by 0.5 cm (95% CI: 0.1 to 0.9), independent of sex, age, socioeconomic level, and ethnicity. SB (accelerometer) was not associated with neck circumference. SB estimated from IPAQ was significantly associated with neck circumference (p<0.05) (Table 4).

**Table 4:** Multilevel regression models for the effect of sedentary behaviors (accelerometer and IPAQ) on body mass index, on neck circumference, and on waist circumference.

Dependent variables	Sedentary behaviors (accelerometer)		Sedentary behaviors (IPAQ)	
	β (95%CI)	p-value	β (95%CI)	p-value
Body mass index (kg/m2)	0.26 (0.08 to 0.44)	0.003	0.12 (-0.02 to 0.26)	0.097
Neck circumference (cm)	0.07 (0.19 to 0.04)	0.203	0.12 (0.02 to 0.21)	0.013
Waist circumference (cm)	0.48 (0.13 to 0.91)	0.002	0.15 (-0.19 to 0.49)	0.389

Multilevel linear regression models, with country as 2nd level, adjusted for sex, age, socioeconomic level, and ethnicity.

β: represents the change in risk factor (body mass index, neck circumference and waist circumference) per 100-minute change in sedentary behaviors.

## Discussion

We aimed to compare self-reported versus accelerometer–measured PA and SB and their associations with body composition in eight Latin American countries. This study found large differences in and low correlations between PA and SB values for IPAQ versus accelerometer measurements. The concordance correlation coefficients between minutes of PA and SB from accelerometry and IPAQ (moderate PA, vigorous PA, MVPA, and SB) were low. MVPA, measured by accelerometer, was negatively associated with body mass index, neck circumference and waist circumference. MVPA estimated from IPAQ was not significantly associated with any of the three anthropometric variables. SB measured by accelerometer was positively associated with body mass index and waist circumference. SB estimated from IPAQ was positively associated only with neck circumference.

The subjective (IPAQ) and the objective (accelerometer) methods used in this study capture different aspects of PA and SB. Accelerometers capture acceleration and laboratory-derived intensity thresholds are used to determine how much time was spent at different intensities of movement (sedentary, light, moderate and vigorous). The questionnaires are designed to provide somewhat similar information to what is captured by accelerometry by asking people to report minutes of activity performed according to intensity as defined by "breathing faster, feeling hotter or sweating". The risk of misinterpretation of intensity coupled with difficulties in remembering the frequency and duration of many activities makes it easy to understand why estimates are so different between methods. Accelerometers capture all movements while worn but may substantially under estimate activity such as cycling that involves less acceleration of the center of gravity. The greater time of self-reported vigorous and moderate intensity PA compared to accelerometer measured MVPA in the current study has been consistently observed in previous studies [9, 40]. Several factors explain this difference. First, accelerometers do not measure activities that do not involve vertical acceleration, such as bicycle movement and upper body movement. In addition, higher-intensity activity sessions (e.g., sports, working out at the gym) are easier to recall and can easily be over-estimated given that many activities involve brief intermittent bursts of activity as well as extended periods with little motion. The observed differences between the two measures increased with higher intensities of activity, similar to previous IPAQ validation studies. Differences and low correlation in PA and SB estimates between accelerometers and questionnaires are evident in comparative studies [13, 40, 41]. The poor Pearson correlation coefficient (i.e., r ≤0.224) observed between accelerometer-measured and self-reported PA in this study is consistent with previous reports in the literature [13, 14, 27, 41].

Precise recall of volume and intensity of PA is not a simple task, as it is influenced by social desirability bias and the subject's inability to recall precise time and intensity of PA [42]. Another strategy to compare procedures that evaluate PA is to assess whether associations with health outcomes are similar. PA measured by accelerometer was significantly associated with body mass index, waist circumference and neck circumference; while PA by self-reported IPAQ was not associated with any body composition variables.

Low correlations and the large differences between mean values of PA measured by accelerometer and self-report are not surprising given that these instruments do not measure the same constructs. IPAQ has questions about time spent on PA by domain (e.g., leisure and active transportation) and intensity (e.g., moderate and vigorous). On the other hand, accelerometry measures continuous motion data or continuous rate of acceleration above defined limits. Despite the challenges and limitations associated with self-reported data on PA and SB, the reality for many large-scale health studies is that this technique is the only executable option given the high cost and logistical complexity of more precise measures such as accelerometry. Accelerometers do not evaluate energy expenditure or intensity in different modalities such as aquatic activity (e.g., swimming, water aerobics) cycling, endurance, and weighted and static exercises, probably leading to a small underestimation of total PA. In addition, data collection, processing, and analysis with accelerometers is substantially more complex than for self-reported questionnaires. PA researchers generally believe that estimates of PA obtained from subjective and objective methods are both useful and complementary, but are not directly comparable. Rather than focusing on which method is “correct”, experts suggest acknowledging that each method contributes unique and complementary information to our understanding of human movement and behavior [9].

The use of self-report remains a good method for large-scale population studies, and it is imperative that these measurements are as accurate and reliable as possible. [43]. The question about sitting time in the last week is a useful metric that has been used in many other surveys [9, 10, 14]. We showed that the correlation of sedentary time estimates obtained using IPAQ and accelerometry was low. The combination of large and relative underestimation and low precision is also likely to significantly reduce the ability to detect associations with outcomes [27, 44]. This may explain publications that report different associations with health outcomes and self-reported and objectively measured sedentary time. Our results contribute to the literature emphasizing the association of SB measured by accelerometer with body composition.

We acknowledge some limitations of this study. The cross-sectional nature of the study limits conclusions regarding of causality. The results may not extrapolate to the total population in the participating Latin American countries. An additional general limitation of accelerometers is that they do not properly capture some activities such as cycling and static exercise. SB defined as ≤100 activity counts/min is a widely accepted standard but does not account for posture and cannot separate sitting from lying down. Therefore, the ≤100 activity counts/min SB classification may either overestimate or underestimate SB and should be used with caution [45]. The major strengths of this study include a large sample size, unique pooling of the data across eight countries from Latin America, concordant assessment of PA and SB using IPAQ and accelerometry, and unique comparable data collection protocols. There are relatively few studies that have used objective measures of PA and SB in Latin American countries, since most international epidemiological investigations use self-reported questionnaires [15]. Our study is the first to compare two methods of assessing PA in different Latin American countries using a comparable methodology.

## Conclusions

IPAQ-reported PA and SB were not significantly associated with health outcomes, while objectively-measured PA and SB were. Self-reported methods may underestimate the strength of some relationships between activity and body composition. The discrepancies observed between accelerometer- and questionnaire-measured PA confirms previous research from other countries. It is important to be aware that these differences between methods also exist in Latin American countries. The results of this study will help with the interpretation of surveillance data collected in the future as well as aiding in the understanding of the associations between movement and health outcomes.

## Supporting information

S1 Table Descriptive analysis (% or mean, 95% CI) of sample profiles concerning demographic, body composition and physical activity variables in participants from the ELANS study. (DOCX)
