# Supplementary material for: Comparison of self-report versus accelerometer – measured physical activity and sedentary behaviors and their association with body composition in Latin American countries
Source: PLoS One. 2020 Apr 28;15(4):e0232420. doi: 10.1371/journal.pone.0232420 (PMC7188285; doi:10.1371/journal.pone.0232420)
Supplement: S1 Table — (DOCX) [file pone.0232420.s001.docx]

**Supporting information**

**S1 Table.** Descriptive analysis (% or mean, 95% CI) of sample profiles concerning demographic, body composition and physical activity variables in participants from the ELANS study.

|  | Argentina | Brazil | Chile | Colombia | Costa Rica | Ecuador | Peru | Venezuela |
| --- | --- | --- | --- | --- | --- | --- | --- | --- |
| N | 270 | 490 | 210 | 294 | 252 | 237 | 308 | 307 |
| Sex (% [95% CI]) | | | | | | | | |
| Male | 43.7  (37.4 to 49.6) | 46.1  (41.4 to 50.6) | 48.1  (41.0 to 54.8) | 50.7  (45.2 to 59.0) | 46.8  (40.9 to 53.2) | 51.1  (42.6 to 59.1) | 47.7  (41.6 to 53.2) | 51.5  (45.6 to 57.3) |
| Female | 56.3  (50.4 to 62.6) | 53.9  (49.4 to 58.6) | 51.9  (45.2 to 59.0) | 49.3  (43.5 to 54.8) | 53.2  (46.8 to 59.1) | 48.9  (42.6 to 55.7) | 52.3  (46.8 to 58.4) | 48.5  (42.7 to 54.4) |
| Age (% [95% CI]) | | | | | | | | |
| 15-19 | 10.4  (6.7 to 14.4) | 11.0  (8.6 to 14.1) | 12.4  (8.1 to 17.1) | 11.2  (7.5 to 15.0) | 13.5  (9.9 to 17.9) | 14.3  (9.7 to 19.0) | 14.0  (10.4 to 17.9) | 13.4  (9.4 to 17.3) |
| 20-34 | 29.6  (24.1 to 34.8) | 37.1  (32.9 to 41.4) | 36.2  (30.5 to 42.4) | 34.7  (29.3 to 40.5) | 35.3  (29.8 to 41.3) | 40.9  (34.6 to 46.8) | 39.6  (33.8 to 45.1) | 43.3  (38.1 to 48.9) |
| 35-49 | 33.7  (28.1 to 39.6) | 28.4  (24.7 to 32.2) | 27.6  (21.9 to 33.8) | 26.9  (21.8 to 32.3) | 31.0  (25.8 to 36.9) | 28.3  (22.8 to 33.8) | 25.6  (20.5 to 30.8) | 27.0  (22.2 to 31.9) |
| 50-65 | 26.3  (20.7 to 31.5) | 23.5  (19.8 to 27.1) | 23.8  (18.1 to 30.0) | 27.2  (22.1 to 32.0) | 20.2  (15.5 to 25.0) | 16.5  (11.8 to 21.5) | 20.8  (16.6 to 25.3) | 16.3  (12.1 to 20.2) |
| M (SD) | 38.9 (14.2) | 37.5 (14.1) | 36.7 (14.2) | 38.0 (14.6) | 36.3 (13.8) | 34.5 (14.2) | 35.3 (14.0) | 34.5 (13.9) |
| Socioeconomic level (% [95% CI]) | | | | | | | | |
| Low | 52.2  (46.3 to 58.5) | 43.7  (39.2 to 48.2) | 37.6  (31.4 to 43.8) | 62.6  (57.1 to 68.0) | 34.1  (28.2 to 40.1) | 46.0  (40.1 to 52.7) | 46.8  (40.9 to 52.9) | 81.4  (77.2 to 85.7) |
| Medium | 43.7  (37.8 to 50.0) | 49.6  (45.3 to 54.1) | 51.4  (44.8 to 58.1) | 32.3  (27.6 to 37.6) | 54.4  (34.2 to 46.8) | 40.5  (34.2 to 46.8) | 29.5  (24.4 to 35.1) | 14.3  (10.1 to 18.2) |
| High | 4.1  (1.9 to 6.3) | 6.7  (4.5 to 9.2) | 11.0  (7.1 to 15.7) | 5.1  (2.7 to 7.5) | 11.5  (7.5 to 15.5) | 13.5  (9.7 to 18.1) | 23.7  (19.2 to 28.9) | 4.2  (2.0 to 6.5) |
| Race/ethnicity (% [95% CI]) | | | | | | | | |
| White | 68.9  (63.7 to 74.4) | 40.8  (36.3 to 45.1) | 29.5  (23.3 to 35.7) | 28.6  (23.5 to 34.0) | 50.4  (44.5 to 56.7) | 3.4  (1.3 to 5.9) | 10.1  (6.8 to 13.3) | 42.0  (36.2 to 47.9) |
| Mixed | 28.1  (22.6 to 33.3) | 20.6  (17.1 to 24.3) | 67.6  (61.4 to 73.8) | 58.5  (52.4 to 63.9) | 35.7  (29.8 to 41.7) | 93.2  (89.9 to 96.2) | 89.0  (85.4 to 92.5) | 48.2  (42.0 to 54.1) |
| Other | 3.0  (1.1 to 5.2) | 38.6  (34.5 to 43.3) | 2.9  (1.0 to 5.2) | 12.9  (9.2 to 17.0) | 13.9  (9.5 to 17.9) | 3.4  (1.3 to 5.5) | 1.0  (0.1 to 2.3) | 9.8  (6.5 to 13.0) |
| BMI (kg/m^2^) | 27.4  (26.7 to 28.1) | 27.1  (26.6 to 27.6) | 27.8  (27.1 to 28.5) | 25.4  (24.9 to 25.9) | 27.7  (26.9 to 28.4) | 26.3  (25.6 to 26.9) | 27.0  (26.4 to 27.5) | 26.7  (26.1 to 27.4) |
| NC (cm) | 36.0  (35.6 to 36.5) | 34.6  (34.2 to 35.0) | 37.3  (36.8 to 37.8) | 35.1  (34.7 to 35.4) | 36.5  (36.0 to 37.0) | 34.9  (34.5 to 35.4) | 35.6  (35.1 to 36.0) | 36.1  (35.7 to 36.6) |
| WC (cm) | 89.7  (87.9 to 91.6) | 87.7  (86.4 to 89.0) | 92.6  (90.8 to 94.4) | 84.7  (83.3 to 86.1) | 92.0  (90.2 to 93.7) | 86.4  (84.9 to 87.9) | 88.1  (86.7 to 89.5) | 87.8  (86.2 to 89.4) |
| Accelerometer (min/day [95% CI]) | | | | | | | | |
| Moderate | 31.5  (28.9 to 34.0) | 33.1  (31.0 to 35.3) | 39.5  (36.4-42.6) | 33.3  (30.7-35.8) | 30.5  (27.5 to 33.5) | 39.1  (35.6 to 42.5) | 34.8  (32.0 to 37.6) | 30.7  (28.3 to 33.1) |
| Vigorous | 0.62  (0.36 to 0.87) | 0.67  (0.49 to 0.85) | 0.82  (0.55 to 1.08) | 0.47  (0.28 to 0.65) | 0.86  (0.55 to 1.16) | 0.72  (0.50 to 0.95) | 0.50  (0.31 to 0.70) | 0.40  (0.23 to 0.56) |
| MVPA | 32.2  (29.5 to 34.8) | 33.8  (31.6 to 36.0) | 40.4  (37.2 to 43.6) | 33.7  (31.1 to 36.4) | 31.5  (28.4 to 34.6) | 39.8  (36.3 to 43.4) | 35.3  (32.5 to 38.2) | 31.1  (28.7 to 33.6) |
| Sedentary | 583.7  (567.8 to 599.9) | 561.5  (550.6 to 572.5) | 560.0  (546.3 to 576.4) | 570.8  (557.9 to 583.3) | 565.0  (550.7 to 580.3) | 573.8  (558.4 to 588.1) | 597.5  (584.3 to 610.8) | 575.1  (562.5 to 588.6) |
| IPAQ (min/day [95% CI]) | | | | | | | | |
| Moderate ^(1)^ | 35.6  (29.4 to 41.8) | 32.7  (28.4 to 37.1) | 52.4  (43.0 to 61.8) | 41.4  (35.3 to 47.6) | 42.9  (36.5 to 49.3) | 60.0  (51.1 to 68.9) | 38.3  (32.5 to 44.1) | 19.9  (16.1 to 23.8) |
| Vigorous | 5.9  (3.7 to 8.0) | 6.1  (4.5 to 7.6) | 10.8  (7.7 to 13.9) | 6.9  (4.9 to 8.8) | 4.5  (2.9 to 6.1) | 10.6  (7.8 to 13.3) | 7.3  (5.3 to 9.4) | 4.9  (3.2 to 6.6) |
| MVPA | 41.5  (34.6 to 48.4) | 38.8  (33.9 to 43.7) | 63.2  (52.6 to 73.8) | 48.3  (41.3 to 55.4) | 47.4  (40.6 to 54.2) | 70.6  (61.1 to 80.0) | 45.6  (39.1 to 52.2) | 24.8  (20.6 to 29.1) |
| Sedentary | 275.1  (257.6 to 293.1) | 213.1  (199.3 to 226.7) | 245.1  (223.6 to 268.1) | 234.8  (216.9 to 253.1) | 227.8  (207.6 to 247.5) | 187.6  (172.9 to 203.5) | 275.0  (258.5 to 292.1) | 20.2  (190.2 to 221.0) |

M: mean; SD: standard deviation; BMI: body mass index; NC: neck circumference; WC: waist circumference: IPAQ: international physical activity questionnaire; MVPA: moderate to vigorous physical activity.

**^(1)^** moderate and moderate-to-vigorous physical activity for international physical activity questionnaire include walking and cycling.
